# High genetic diversity in the offshore island populations of the tephritid fruit fly *Bactrocera dorsalis*

**DOI:** 10.1186/s12898-016-0101-0

**Published:** 2016-10-13

**Authors:** Chunyan Yi, Chunyan Zheng, Ling Zeng, Yijuan Xu

**Affiliations:** Laboratory of Insect Ecology, Department of Entomology, South China Agricultural University, Guangzhou, 510640 China

**Keywords:** Oriental fruit fly, Population genetic structure, Mitochondrial DNA, Microsatellite, Genetic diversity, Island isolation

## Abstract

**Background:**

Geographic isolation is an important factor that limit species dispersal and thereby affects genetic diversity. Because islands are often small and surrounded by a natural water barrier to dispersal, they generally form discrete isolated habitats. Therefore, islands may play a key role in the distribution of the genetic diversity of insects, including flies.

**Results:**

To characterize the genetic structure of island populations of *Bactrocera dorsalis*, we analyzed a dataset containing both microsatellite and mtDNA loci of *B. dorsalis* samples collected from six offshore islands in Southern China. The microsatellite data revealed a high level of genetic diversity among these six island populations based on observed heterozygosity (Ho), expected heterozygosity (H_E_), *Nei’s* standard genetic distance (*D*), genetic identity (*I*) and the percentage of polymorphic loci (PIC). These island populations had low *F*_ST_ values (*F*_ST_ = 0.04161), and only 4.16 % of the total genetic variation in the species was found on these islands, as determined by an analysis of molecular variance. Based on the mtDNA COI data, high nucleotide diversity (0.9655) and haplotype diversity (0.00680) were observed in all six island populations. *F*-statistics showed that the six island populations exhibited low or medium levels of genetic differentiation among some island populations. To investigate the population differentiation between the sampled locations, a factorial correspondence analysis and both the unweighted pair-group method with arithmetic mean and Bayesian clustering methods were used to analyze the microsatellite data. The results showed that Hebao Island, Weizhou Island and Dong’ao Island were grouped together in one clade. Another clade consisted of Shangchuan Island and Naozhou Island, and a final, separate clade contained only the Wailingding Island population. Phylogenetic analysis of the mtDNA COI sequences revealed that the populations on each of these six islands were closely related to different populations on mainland China.

**Conclusions:**

Our study suggests that these island populations have high genetic diversity, experience frequent gene flow and exhibit low or medium levels of genetic differentiation among some island populations. Therefore, the geographic isolation of the six islands does not appear to be a major dispersal barrier to *B. dorsalis*. Such knowledge is helpful for a better understanding of evolutionary processes of the species of island populations.

**Electronic supplementary material:**

The online version of this article (doi:10.1186/s12898-016-0101-0) contains supplementary material, which is available to authorized users.

## Background

Genetic diversity is a critical component of biodiversity and affects the survival and evolution of species [1, 2]. Geographic isolation is an important factor that limits species dispersal and affects the genetic diversity of species [3, 4]. By definition, islands are smaller than continents, surrounded by water, and therefore form discrete habitats isolated from other terrestrial habitats [5, 6]. Generally, the water surrounding islands acts as a geographic barrier to dispersal that limits gene flow both between island populations and between island and mainland populations. Consequently, the genetic diversity of insular species tends to be more complex due to multiple factors that include the natural dispersal distance of flight-capable species and dispersal that is mediated by human activity. In such special cases, island populations have lower genetic diversity than do mainland populations due to founder effects/bottlenecks and continued isolation from the mainland [3, 7–10]. To maintain a population’s fitness and adapt to an island environment, a population may lose genetic diversity [11]. Previous bottlenecks or continued isolation from the mainland can also decrease genetic diversity. Moreover, the loss of genetic diversity in island populations can often be caused by founder effects, breeding rates, and dispersal ability, among other factors. However, the island populations of some species, i.e., mammals and birds, have higher levels of genetic diversity than do mainland populations [8, 11, 12].

*Bactrocera dorsalis* (Hendel) (Diptera: Tephritidae), the oriental fruit fly, is a quarantined pest that is found worldwide, is highly fecund and highly adaptable. This fly species causes serious economic losses to fruit production in tropical and subtropical areas [13–16]. The original source of *B. dorsalis* populations has been reported to be from tropical or subtropical Asia [17]. To date, *B. dorsalis* has spread over Asia and to many regions around the Pacific, including Hawaii [18]. *Bactrocera dorsalis* was first recorded in Taiwan in 1911 but has since spread to many other provinces in China [19, 20]. Wan et al. [20] examined the genetic diversity and genetic differentiation of *B. dorsalis* in the Chongqing region in China. Their results indicated that the height of these mountains in this region was insufficient to prevent the long-distance dispersal of *B. dorsalis* and suggested that there would be a high frequency of gene flow among fly populations. However, Li et al. [16] demonstrated that geographic isolation from mountains and canyons distributed across China, Vietnam and Thailand slowed the dispersal of *B. dorsalis* and has resulted in genetic differentiation between these regions. To date, no studies have explored the genetic diversity of *B. dorsalis* on islands. In this study, we hypothesized that six island populations of *B. dorsalis* in South China would have lower levels of genetic diversity due to their isolation from the mainland. Furthermore, the isolation of islands may also result in high levels of genetic differentiation between different island populations. In this study, we generated and analyzed a dataset of both microsatellite and mtDNA loci from *B. dorsalis* samples collected from six offshore island populations in South China to estimate the genetic divergence and dispersal ability of these flies. Our results may also suggest possible strategies for the control of this species based on their dispersal patterns.

## Methods

### Sample collection

**Fig. 1:**
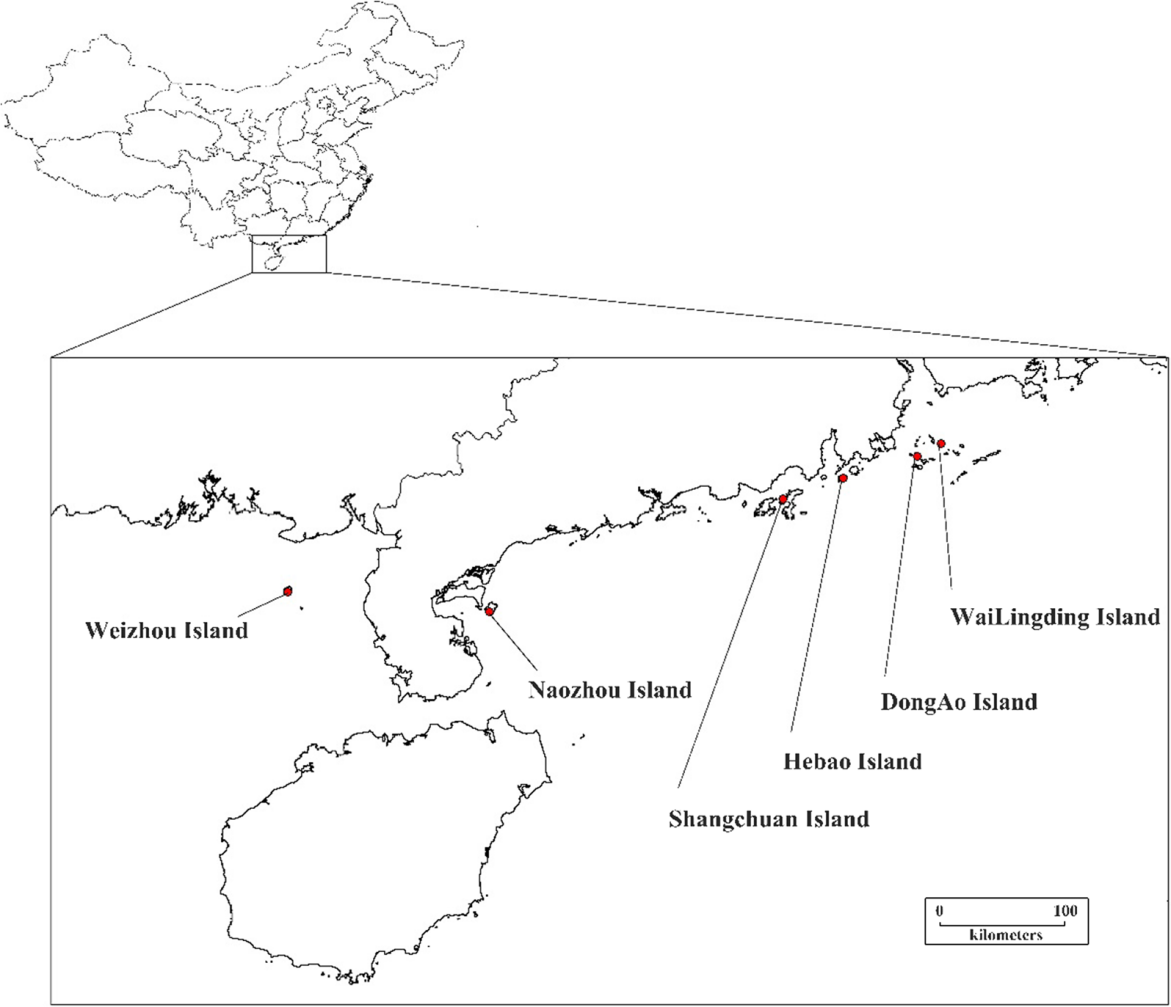
Locations of the six island populations of *Bactrocera dorsalis* examined in this study. The map for sample distribution was generated by DIVA-GIS version 7.1

*Bactrocera dorsalis* were sampled from six offshore islands in South China (Fig. 1), and the longitude and latitude information of all locations were recorded. No permissions were required to collect these samples from the field. The map for sample distribution was generated by DIVA-GIS version 7.1. Shangchuan Island, Hebao Island, Dong’ao Island and Wailingding Island are located in Guangdong Province, and Naozhou Island and Weizhou Island are located in Guangxi Province. A number of fruits, including mango, banana and papaya, are cultivated on the islands. However, there is an exceptionally low population density of *B. dorsalis* on the islands. Male *B. dorsalis* individuals were captured using methyl eugenol-baited traps on each island. After collection, the samples were preserved in 90 % ethanol prior to DNA extraction.

### PCR amplification

DNA from individual specimens was extracted using the TIANamp Genomic DNA Kit (Qiagen, Hilden, Germany). In total, DNA was extracted from 20 flies from each island. Eight polymorphic microsatellite loci (MS4, MS6, MS12A, 4.3A, 6.8A, 4.6A, Ccmic32 and Bo-D48) were analyzed, all of which were previously developed [14, 21–23] (see Additional file 1: Table S1). Primers were synthesized by Sangon Biotech Co. Ltd (Shanghai, China). PCR reactions were performed in 25 µL volumes containing 2 µL of DNA extract (40 ng/µL), 2.5 µL of 10× PCR buffer (containing Mg^2+^), 2 µL of a dNTP mixture (10 mmol/L), 1.5 µL of each primer (10 mmol/L), 0.2 µL of Taq DNA polymerase (5 U/µL) and 15.3 µL of ddH_2_O. Cycling conditions for amplification were as follows: 3 min at 94 °C followed by 35 cycles of 30 s at 94 °C, 30 s at 48 °C, 45 s at 72 °C, and an extension of 5 min at 72 °C.

The PCR-amplified products were separated using a DNF-900 High Sensitivity Large Fragment Analysis Kit in a fragment analyzer (Advanced Analytical Technologies, Inc., USA). Alleles were scored using PROSize 2.0 (Advanced Analytical Technologies, Inc., USA).

A 759-bp fragment of the cytochrome oxidase subunit I (COI) gene was amplified using primers (COI-F: CAACATTTATTTTGATTTTTTGG; COI-R: TCCATTGCACTAATCTGCCATATTA; synthesized by Sangon Biotech Co., Ltd (Shanghai, China)) and following the method described by Tang et al. [24, 25]. The PCR reaction volumes were the same as the microsatellite DNA amplification volumes, except the annealing temperature duration was 45 s.

### Microsatellite data

Deviations from Hardy–Weinberg proportions and linkage disequilibrium were calculated using Genepop version 5.0 (http://www.wbiomed.curtin.edu.au/Genepop/Genepop_op1.html) [26]. We estimated null allele frequencies for each locus using the expectation maximization (EM) algorithm method in the FREENA software (http://www.montepllier.inra.fr/URLB) [27]. Population genetic parameters included the observed number of alleles (NA), the effective number of alleles (*N*_*E*_), Shannon’s information index (*I*), observed heterozygosity (*H*_*o*_), expected heterozygosity (*H*_*E*_), the percentage of polymorphic loci (*P*), Nei’s genetic diversity (Nei’s), gene flow (*N*_*em*_), Nei’s original measures of genetic identity (*I*) and genetic distance (*D*) for paired populations. These parameters were then analyzed using Popgene Version 1.3.1 [28]. The population genetic structure was analyzed with STRUCTURE v.5.0 [29], which uses a Bayesian Markov Chain Monte Carlo (MCMC) method to analyze the numbers of K genetic clusters. We ran K values from 1 to 6 using an admixture model and an allele frequencies correlated model. The first 100,000 repetitions were discarded as burn-in, and 10,000 MCMC repetitions were then run. Multiple runs are used to check consistency between runs. Then, the results after running STRUCTURE were then uploaded to the Structure Harvester website (http://www.taylor0.biology.ucla.edu/structureHarvester/#). Structure Harvester was used to calculate six iterations per K-value and obtained the best K-value using the ΔK method of Evanno. [30]. *Nei’s* genetic distance (D) for the six island populations was reconstructed for the population phylogenetic tree using the unweighted pair-group method with arithmetic mean (UPGMA) in the tools for population genetic analyses (TFPGA) program [31]. We used a multidimensional factorial correspondence analysis (FCA) within the GENETIX program to identify clusters of individuals with similar genotypes based on allele frequencies and genotype [32].

Isolation by distance was measured using the TFPGA software. Matrix correlations using pairwise genetic distance versus geographic distance were estimated and significance was determined using the Mantel test.

Analysis of molecular variance (AMOVA) was performed with ARLEQUIN ver. 3.5 to partition the genetic variance within and among populations based on the numbers of different alleles (*F*_*ST*_) and the sum of squared size differences [33].

### Mitochondrial data

Published COI sequences of *B. dorsalis* from mainland China populations and mainland populations from other countries (Cambodia, Mandalay, Laos and America) were downloaded from NCBI [16, 34] to characterize the genetic relationships between these mainland populations and the studied six island populations in South China. We cut and joined the amplification sequences using one pair of primers with DNAStar and manually corrected any obvious errors. Next, we aligned all sequences using MEGA6 software [35]. A population phylogenetic tree was reconstructed using the UPGMA method in MEGA6 based on genetic distances. Haplotype diversity (*Hd*), the average number of differences (*k*), and nucleotide diversity (*π*) were calculated for each population using DnaSP 5.0 [20, 36].

The fixation index (pairwise *F*_*ST*_) and gene flow (*N*_*em*_) were estimated using ARLEQUIN ver. 3.5 [33]. Generally, gene exchange leading to low genetic differentiation between populations occurs when *N*_*em*_ > 4 [37]. The significance level was assessed using 1000 permutations. The index was interpreted as follows: a low degree of genetic differentiation (0 ≤ *F*_*ST*_ < 0.05); a medium degree of genetic differentiation (0.05 ≤ *F*_*ST*_ < 0.15); a high degree of genetic differentiation (0.15 ≤ *F*_*ST*_ ≤ 0.25); and a very high degree of genetic differentiation (*F*_*ST*_ > 0.25) [38, 39].

## Results

### Microsatellite analysis

#### Hardy–Weinberg and genetic diversity

All loci and samples significantly deviated from Hardy–Weinberg proportions (P < 0.01). The average values of *F*_*IS*_ for each locus in each population ranged from 0.0365 to 0.8353, which indicated that there were deficiencies in heterozygotes. Over all populations and loci, the locus MS12A for the DAD population had the highest null allele frequency (*N*_*a*_ = 0.41419). The null allele frequency of the other loci and populations ranged from 0 to 0.37780. The presence of null alleles was the primary contributor to the deficiencies in heterozygotes and the departure from the Hardy–Weinberg equilibrium. There was no significant linkage disequilibrium between pairs of loci for all populations (P > 0.05). Therefore, the eight loci were inherited independently.

In total, 107 alleles were observed for eight microsatellite loci among the six island populations, and the number of alleles per locus ranged from 7 (Locus 4.3A) to 19 (Locus 6.8A), with an average of 13.375 (Additional file 1: Table S2). The expected heterozygosity (*H*_*E*_) of each locus ranged from 0.5397 to 0.8806, and the observed heterozygosity (*H*_*O*_) ranged from 0.1008 to 0.6441. The observed number of alleles per island population ranged from 5.75 (Dong’ao Island) to 8 (Wailingding Island) (Table 1), with an average of 7.3214. The expected heterozygosity per population ranged from 0.7532 (Wailingding Island) to 0.2442 (Hebao Island). The results showed that the six island populations all exhibited high levels of genetic diversity. The Hebao Island population exhibited a lower level of genetic diversity than did the other five island populations. Nei’s genetic diversity index for the Hebao Island population was 0.1769, which was far lower than the other five island populations (Table 1).

**Table 1:** Indices of genetic diversity of *Bactrocera dorsalis* populations from six islands based on microsatellite data

Population	*N*_*A*_	*N*_*E*_	*I*	*H*_*O*_	*H*_*E*_	*Nei’s*	*Np*	*P* (%)
Dong’ao Island	5.7500 ± 2.1876	3.7076 ± 1.8766	1.3450 ± 0.4904	0.2970 ± 0.2371	0.6740 ± 0.1850	0.6571 ± 0.1804	8	100
Wailingding Island	8.0000 ± 2.3905	4.4046 ± 1.8152	1.6184 ± 0.4118	0.4313 ± 0.2359	0.7532 ± 0.1131	0.7344 ± 0.1103	8	100
Hebao Island	7.1250 ± 2.1671	3.4046 ± 1.6079	1.3886 ± 0.4488	0.3355 ± 0.2442	0.2442 ± 0.1769	0.1769 ± 0.1725	8	100
Shangchuan Island	7.6250 ± 2.8754	3.8322 ± 1.7547	1.4875 ± 0.5302	0.3500 ± 0.3218	0.6697 ± 0.1983	0.6869 ± 0.1933	8	100
Weizhou Island	6.8750 ± 2.7999	3.4946 ± 1.5458	1.4143 ± 0.4487	0.2812 ± 0.2103	0.6796 ± 0.1494	0.6627 ± 0.1457	8	100
Naozhou Island	7.8750 ± 2.7999	4.4092 ± 1.9838	1.6249 ± 0.4329	0.4016 ± 0.2906	0.7546 ± 0.1072	0.7356 ± 0.1046	8	100
Mean	7.3214 ± 0.8192	3.8755 ± 0.4386	1.4798 ± 0.1192	0.3494 ± 0.0583	0.6292 ± 0.1926	0.6089 ± 0.2143	8	100

*N*_*A*_ Observed number of alleles; *N*_*E*_ Effective number of alleles [42]; *I* Shannon’s information index [43]; *Nei’s* Nei’s gene diversity; *H*_*O*_ observed heterozygosity; *H*_*E*_ expected heterozygosity; *Np* number of polymorphic loci; *P* percentage of polymorphic loci

#### Population genetic structure

**Fig. 2:**
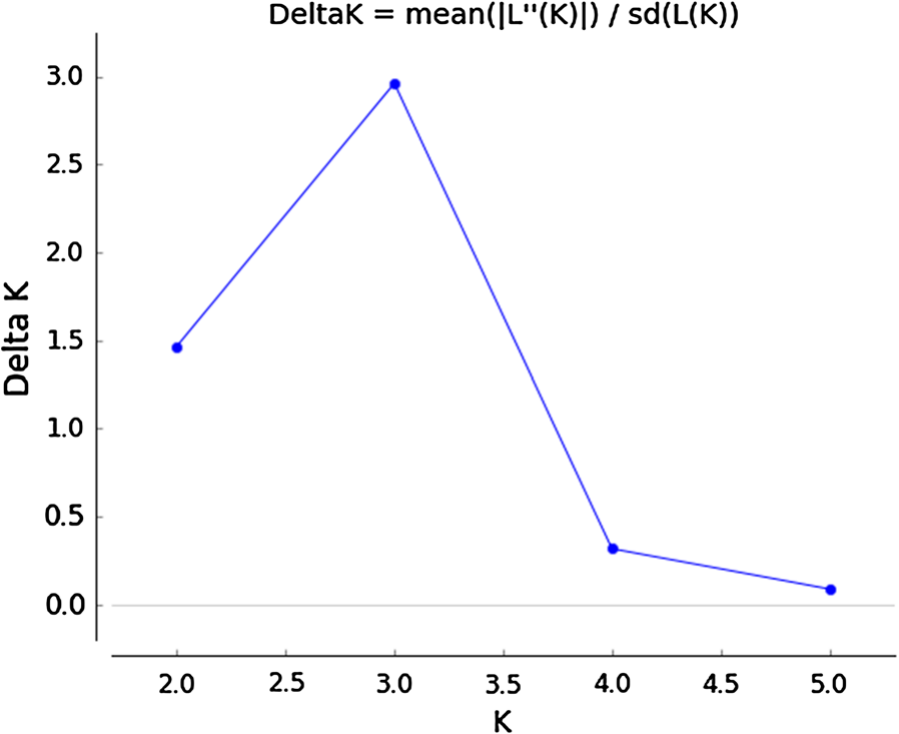
Line graph of genetic cluster (K) vs. Delta K

**Fig. 3:**
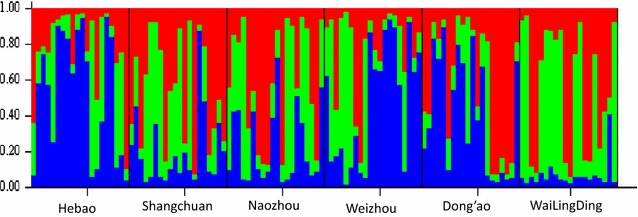
Population genetic structure of *B. dorsalis* based on microsatellite data assigned to three clusters. Each individual is represented by a *vertical bar*

We analyzed the genetic structure of the different *B. dorsalis* populations using the Bayesian clustering analysis method from the STRUCTURE software. The results showed that the best possible ΔK was 3 (Fig. 2). This finding suggested that the six island populations were divided into three clusters based on the allele frequencies of the geographic populations. Hebao Island, Weizhou Island and Dong’ao Island were clustered into one branch (highlighted in blue). Shangchuan Island and Naozhou Island were grouped into another branch (highlighted in red). The last branch consisted only of the Wailingding Island population (highlighted in green) (Fig. 3).

**Fig. 4:**
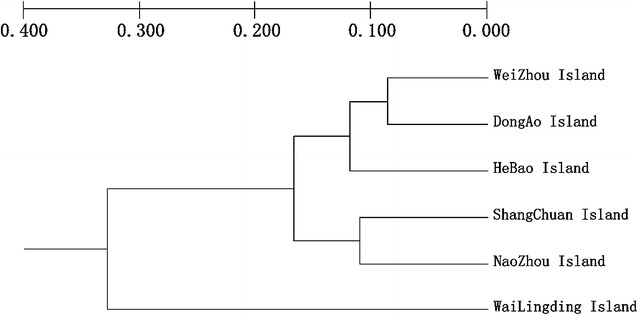
Phylogenetic analyses of *B. dorsalis* populations from six islands based on microsatellite variations using the unweighted pair-group method with arithmetic mean (UPGMA)

We used TFPGA software to construct an UPGMA population phylogenetic tree, and the results showed that the six island populations could be divided into three groups (Fig. 4). Hebao Island, Weizhou Island, and Dong’ao Island were classified into one group; Shangchuan Island and Naozhou Island were classified into another group; and Wailingding Island was classified into the final group. The results from the phylogenetic tree using UPGMA based on microsatellite data were consistent with those obtained from the STRUCTURE analysis.

**Fig. 5:**
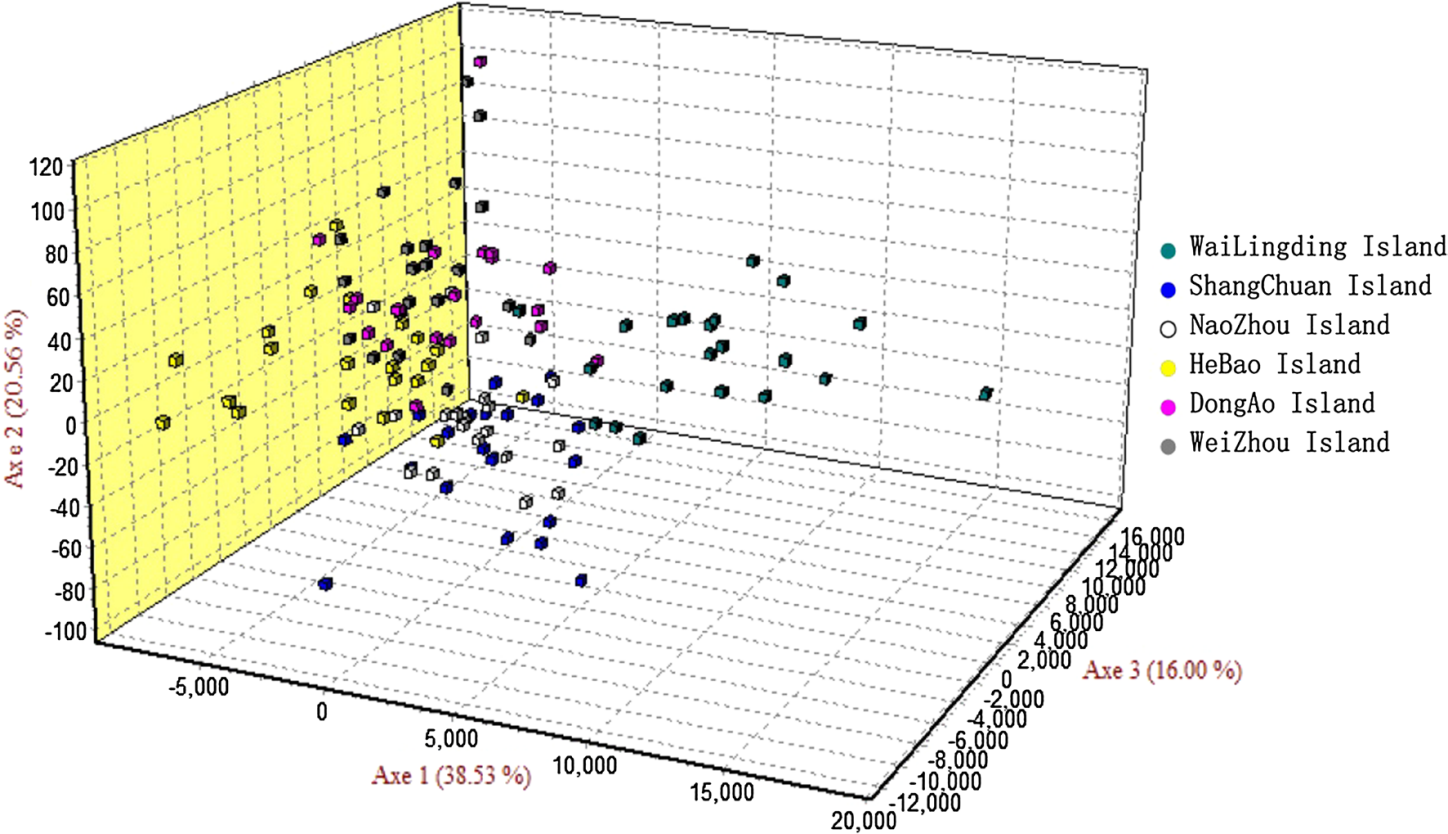
Three-dimensional correspondence analysis (FCA) of microsatellite genotypes from six offshore island populations

We analyzed the genetic divergence among populations based on the individual genotypes and constructed the three-dimensional FCA shown in Fig. 5. According to this graph, the results of the FCA were the same as the results from STRUCTURE and the phylogenetic tree.

Hierarchical *F*-statistics were estimated for all populations as a single group and for all populations partitioned into three groups based on the results of the Bayesian clustering analysis. AMOVA results indicated that genetic variation primarily contributed to variation among individuals within populations and to variation within individuals (Table 2). The genetic variation that was divided into one group and three groups was 48.6 % (*F*_*IT*_ = 0.52765, P < 0.001) and 47.9 % (*F*_*IS*_ = 0.50631, P < 0.001) of the total variation, respectively. In addition, within individual variation accounted for 47.23 % (*F*_*IT*_ = 0.52765, P < 0.01) and 46.75902 % (*F*_*IT*_ = 0.53241, P < 0.01) of the total variation for the one group and three groups analyses, respectively. Most of the total genetic variation was explained by the variation among individuals within populations.

**Table 2:** AMOVA results based on microsatellite genotypes

Source of variation	d.f.	Variance components	Percentage of variation	Fixation indices
Among populations	5	0.12261 Va	4.16	F_ST_ = 0.04161**
Among individuals within populations	114	1.43202 Vb	48.6	F_IS_ = 0.50715**
Within individuals	120	1.39167 Vc	47.23	F_IT_ = 0.52765**
Among groups	2	0.13294 Va	4.46153	FCT = 0.04462**
Among populations within groups	2	0.02457 Vb	0.82472	FSC = 0.00863
Among individuals within populations	114	1.42888 Vc	47.95	FIS = 0.50631**
Within individuals	120	1.39325 Vd	46.75902	FIT = 0.53241**

As shown in Table 3, *Nei’s* standard genetic distance (*D*) (below the diagonal) among six island populations ranged from 0.0853 to 0.4021 and *Nei’s* genetic identity (I) (above the diagonal) varied from 0.6689 to 0.9182. The population genetic identity (I) between the Wailingding island population and the Weizhou island population was 0.6689, which was the minimum among all paired island populations. Accordingly, there was high genetic distance, which was 0.4021 between these paired island populations (Table 3).

**Table 3:** Nei’s original measures of genetic identity (above diagonal) and genetic distance (below diagonal)

Population	Hebao	Shangchuan	Naozhou	Weizhou	Dong’ao	Wailingding
Hebao	****	0.8095	0.8433	0.8897	0.8883	0.6752
Shangchuan	0.2113	****	0.8968	0.8238	0.8631	0.7691
Naozhou	0.1704	0.1090	****	0.8889	0.8539	0.7570
Weizhou	0.1169	0.1938	0.1177	****	0.9182	0.6689
Dong’ao	0.1184	0.1473	0.1580	0.0853	****	0.7410
Wailingding	0.3928	0.2625	0.2784	0.4021	0.2998	****

**Fig. 6:**
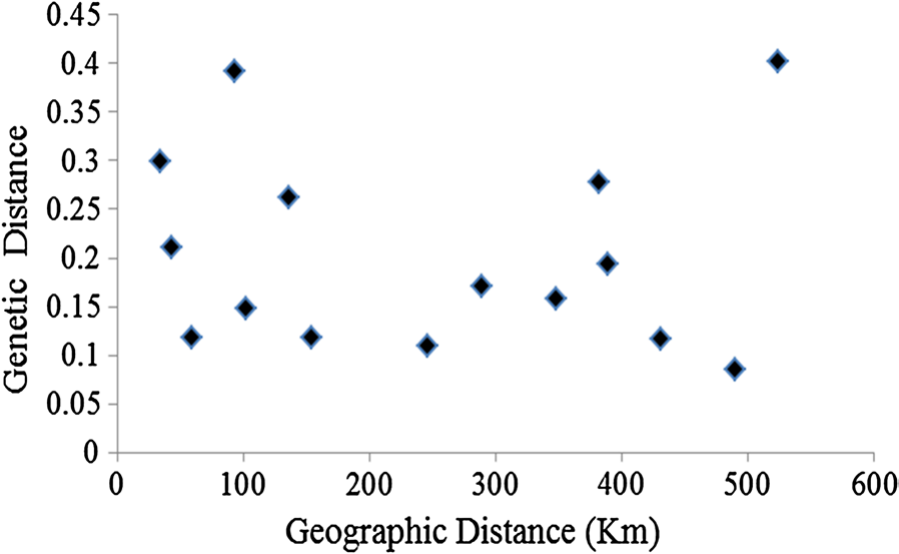
The relationship between genetic distance and geographic distance of island populations

The relationship between genetic distance and geographic distance was determined among island ranges. The result showed that there was no relationship between genetic distance and geographic distance (Fig. 6; R^2^ = 0.0027, P = 0.855).

### Mitochondrial analysis

We obtained 120 sequences from COI with a length of 759 bp. In total, 66 different mitochondrial haplotypes were detected in the six island populations. All populations on the six islands had high levels of nucleotide (0.0068) and haplotype diversity (0.9665). Haplotype diversity (*Hd*) for each population ranged from 0.889 to 0.995. The average number of differences (*k*) ranged from 3.895 (Wailingding Island) to 6.553 (Naozhou Island). Nucleotide diversity (*π*) for each population ranged from 0.513 % (Wailingding Island) to 0.863 % (Naozhou Island) (Table 4).

**Table 4:** Haplotype diversity (Hd), average number of differences (k) and nucleotide diversity (π) for each population

Population	N	Haplotype (frequency)	*Hd*	*π*	*k*
Dong’ao Island	20	H1(1), H2(1), H3(5), H4(2), H5(5), H6(1), H7(1), H8(1), H9(1), H10(1), H11(1)	0.889 ± 0.049	0.00633	4.805
Wailingding Island	20	H3(3), H5(1), H10(1), H13(1), H19(4), H23(2), H57(1), H105(1), H142(1), H143(1), H144(1), H145(1), H146(1), H147(1)	0.947 ± 0.034	0.00513	3.895
Hebao Island	20	H3(2), H5(4), H8(1), H13(1), H19(1), H25(1), H74(1), H77(1), H102(1), H103(1), H104(1), H105(1), H106(1), H107(1), H108(1), H109(1)	0.963 ± 0.033	0.0062	4.705
Shangchuan Island	20	H3(2), H5(2), H8(1), H11(1), H13(1), H19(2), H37(1), H77(2), H105(1), H133(1), H134(1), H135(1), H136(1),H137(1), H138(1), H139(1)	0.979 ± 0.021	0.00659	5.005
Weizhou Island	20	H3(2), H5(2), H44(1), H62(1), H77(1), H107(1), H129(1), H140(1), H142(1), H148(1), H149(1), H150(1), H151(1), H152(1), H153(2), H154(1), H155(1)	0.984 ± 0.020	0.00689	5.232
Naozhou Island	20	H5(2), H8(1), H25(1), H52(1), H77(1), H119(1), H120(1), H121(1), H122(1), H123(1), H124(1), H125(1), H126(1), H127(1), H128(1), H129(1), H130(1), H131(1), H132(1)	0.995 ± 0.018	0.00863	6.553
Total	120	66	0.9665 ± 0.008	0.00680	5.112

*N* sample size; *Hd* haplotype diversity; *π* nucleotide diversity; *k* average number of differences

There was genetic divergence between the six island populations, as estimated by population pairwise *F*_*ST*_ significance tests. The *F*_*ST*_ values between paired groups ranged from −0.03194 to 0.05413. The Dong’ao Island population had a low degree of differentiation with the Wailingding Island, Hebao Island and Shangchuan Island populations (0.01024 ≤ *F*_*ST*_ ≤ 0.01895). However, Dong’ao differentiated from the Weizhou and Naozhou Island populations (0.05065 ≤ *F*_*ST*_ ≤ 0.05413, P < 0.05) (Table 5). Hebao Island had no differentiation from the Wailingding, Shangchuan and Naozhou Island populations (−0.00179 ≤ *F*_*ST*_ < 0) (Table 5). There were low and medium levels of differentiation among some paired island populations. The value of gene flow (*N*_*em*_) between each pair of populations was over four, which suggested that there was a full exchange of genes between the six island populations. The isolation of the islands did not prevent gene flow between the populations. These results were consistent with the microsatellite data.

**Table 5:** *F*_*ST*_ values (below diagonal) and *N*_*em*_ (above diagonal) between the six island populations of *Bactrocera dorsalis*

Population	Dong’ao	Wailingding	Hebao	Shangchuan	Weizhou	Naozhou
Dong’ao	****	48.3333	39.8018	25.8889	8.7377	9.3713
Wailingding	0.01024	****	inf	inf	42.6287	14.8611
Hebao	0.01241	−0.01058	****	inf	80.6048	inf
Shangchuan	0.01895	0	−0.03194	****	178.3258	inf
Weizhou	0.05413*	0.01159	0.00616	0.0028	****	134.2085
Naozhou	0.05065*	0.03255*	−0.00179	−0.00089	0.00371	****

Data with asterisks indicate significant difference (P < 0.05)

**Fig. 7:**
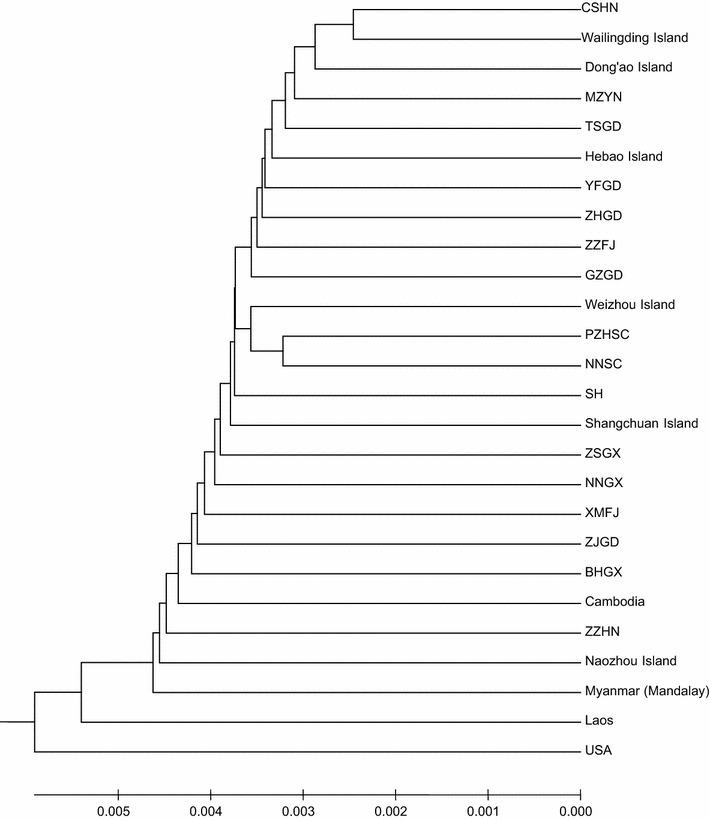
Phylogenetic analyses of *B. dorsalis* populations from six islands and other locations [10, 21] based on variations in mitochondrial DNA using the unweighted pair-group method with arithmetic mean (UPGMA). CSHN (Changsha, Hunan), MZYN (Mengzi, Yunnan), TSGD (Taishan, Guangdong), YFGD (Yunfu, Guangdong), ZHGD (Zhuhai, Guangdong), ZZFJ (Zhangzhou, Fujian), GZGD (Guangzhou, Guangdong), PZHSC (Panzhihua, Sichuan), NNSC (Ningnan, Sichuan), SH (Shanghai), ZSGX (Zhongshan, Guangxi), NNGX (Nanning, Guangxi), XMFJ (Xiamen, Fujian), ZJGD (Zhanjiang, Guangdong), BHGX (Beihai, Guangxi), and ZZHN (Zhengzhou, Henan) were all from China

The topology of the *B. dorsalis* population maximum likelihood tree suggested that there were no independent groups in the island populations. In the phylogenetic tree, these six island populations of Southern China were clustered in one branch with different mainland populations of China (Fig. 7). We also found that the Shangchuan and Naozhou island populations were more closely related to populations from Cambodia and Myanmar than to populations from Laos and the United States (Figs. 5, 7).

## Discussion

### Null alleles

Null alleles, geographic factors and likely founder effects among populations caused heterozygote deficiencies and emerged as significant departures from the Hardy–Weinberg equilibrium [40]. In this study, the frequency of null alleles in an isolated microsatellite of *Bactrocera dorsalis* was high and exceeded 0.4 only among locus 6.4. We checked the heterozygotes and found significant heterozygote deficiencies, except for locus MS12A, in several island populations.

### Genetic diversity

Population genetic diversity is a product of evolutionary change over several generations [41]. Thus, population genetic diversity reflects the ability of populations to adapt to local environments [42]. The percentage of polymorphism, the level of heterozygosity, the number of alleles, nucleotide diversity and haplotype diversity were used to estimate population genetic diversity. Our research showed that populations of the oriental fruit fly from islands in South China had high levels of genetic diversity and were closely related to populations from mainland China based on COI data analysis. Using the mtDNA COI from five Yunnan province populations of *B. dorsalis,* Shi et al. [43] showed that the average haplotype diversity and nucleotide diversity of the five populations were 0.9786 and 0.9038 %, respectively. Thus, the five Yunnan populations had higher levels of genetic diversity than did the island populations examined in this study. Analysis of the genetic diversity of *B. dorsalis* from China, Laos, and Thailand with microsatellite markers showed that the average *Nei’s* genetic diversity and Shannon’s information index were 0.6464 and 0.7870, respectively. The average percentage of polymorphic loci in all populations was 94.45 %. This finding suggests that mainland South China has high levels of population genetic diversity [44].

However, many studies have shown that the gene flow of mammals, birds and other species from islands is blocked or reduced due to isolation, which results in lower levels of genetic diversity of island populations compared to mainland populations [8, 14, 45]. Isolated islands have discrete boundaries that are generally thought to reduce migration between populations on islands [3]. Long-term bottlenecks, founder effects, genetic drift and inbreeding may all reduce genetic diversity [46, 47]. Mitochondrial and microsatellite data have been previously used to estimate the genetic structure of *Apis mellifera* populations from the Canary Islands [7]. The results showed that there was a lower level of genetic variation based on the average number of alleles and heterozygosity in populations on the Canary Islands than mainland populations in Iberia and Morocco [48]. Boessenkool et al. reported that natural populations of robins on the Breaksea and Nukuwaiata Islands have lower levels of genetic diversity than larger mainland populations. In addition, some alleles have been lost, which is a result of bottlenecks and isolation from the mainland [49]. Jensen et al. [3] reported that the genetic diversity of house sparrows along a coastal latitudinal gradient from middle to Northern Norway tended to decrease in island populations compared to mainland populations. However, in our study, the results showed that the genetic diversity of *B. dorsalis* from island populations was high. The geographic isolation imposed by the sea may not hinder gene exchange. There are many reasons for high genetic diversity among island populations, including the dispersal ability of this species and both environmental and anthropogenic activities that cause frequent contact with mainland populations. The six islands have been isolated for a long period of time, and most of the islands originated from the accumulation of volcanic matter. As the islands developed, commercial activities on the islands increased, which has incidentally promoted the spread of species throughout the islands. *B. dorsalis* can use most commercial plants and fruits as host plants, and the flies can disperse between the mainland and islands by relying on the wind. According to the phylogenetic tree analysis, there were two main routes of invasion from inland China. One route was from Southeast China, and the other route was from Southwest China [50]. However, whether *B. dorsalis* regularly makes migratory flights from the nearby mainland to these islands is unclear.

### Genetic divergence

For COI data, the value of *N*_*em*_ among populations all exceeded 4 (*N*_*em*_ > 4), which showed that among populations there was a high levels of gene flow and a low and medium level of differentiation among some paired island populations. These results were consistent with the results from our microsatellite data that shows a low *Nei’s* standard genetic distance (D) and a high genetic identity (I). Pairwise *F*_*ST*_ values indicated that there was a medium degree of population differentiation between Weizhou Island and Naozhou Island. The microsatellite data indicated that population genetic variation was mostly partitioned among populations. We divided the six island populations into three groups, which were supported by the FCA. Our results were also similar to the levels of population differentiation between mainland China populations. Wan et al. [20] analyzed the genetic diversity of *B. dorsalis* from six populations in Qiongqing using eight microsatellite loci. The results showed that the Qiongqing populations had low levels of population differentiation. Yao et al. [51] also examined the genetic relationships among populations from Fujian, Hainan, Guangdong, Yunnan and Sichuan provinces. Except for the Fujian population, all populations had low levels of population differentiation because of the proximity between provinces. Using mtDNA from 25 populations on the China mainland, flies from mainland China had lower levels of genetic divergence than those from Thailand, Japanese and American populations [50]. Li et al. [16] reported that the average Nei’s standard genetic distance was 0.8049 and 0.9397, for South China and Southeast Asia populations, respectively. Li et al. [16] also reported that *F*_*ST*_ was 0.25 between South China and Southeast Asia populations, which suggested that these populations had genetic divergence due to geographic isolation. Our study suggested that island *B. dorsalis* populations had high levels of genetic diversity and a low or medium level of differentiation among some paired island populations and the genetic distance of pairing populations had no correlation with geographic isolation (R^2^ = 0.0027, P = 0.855). There are several possible explanations for this pattern. First, Tephritid fruit flies are capable fliers that can fly more than 25 km under windless conditions [52, 53]. The distance between each island and the mainland ranged from 8.2 to 38.4 km. The distance between islands ranged from 32.4 to 519.7 km. Hebao Island, Wailingding Island and Dong’ao Island are located in Zhuhai province and are closer in proximity than are Weizhou Island and Naozhou Island. Thus, Hebao Island populations were barely differentiated from the Wailingding Island, Shangchuan Island and Naozhou Island populations. However, there was a medium degree of genetic differentiation between Dong’ao Island and Weizhou Island populations and between Dong’ao Island and Naozhou Island populations. *Bactrocera dorsalis* can migrate to and from islands on its own or with the aid of typhoons [52]. The isolation of islands by water limits gene flow. However, in our study, the islands did not appear to restrict gene flow: the population genetic diversity and differentiation on the islands were similar to values observed for mainland populations. Second, based on the results of our study, commercial activities between the island and the mainland have affected the gene pool and diversity of species. These six islands all have suitable host plants for *B. dorsalis,* such as banana, pawpaw and others. Many tropical fruits, such as guava, carambola, and mango, are imported and exported from the mainland to the islands. *Bactrocera dorsalis* can lay eggs on these fruits, and the infested fruits can subsequently be transported to other areas. Therefore, gene flow is likely frequent between islands. The low levels of genetic diversity are also likely caused by the short distance between the islands and the mainland.

## Conclusions

This study showed that island isolation may not significantly influence the genetic diversity of tephritid fruit flies. We have found that offshore island populations of *B. dorsalis* have relatively high levels of genetic diversity, whereas populations from offshore islands exhibited low genetic differentiation.

## Additional file

**Additional file 1: Table S1.** Primer sequences and PCR characteristics of 8 microsatellite loci. **Table S2.** Indices of genetic diversity and gene flow inferred from microsatellite data.
