# Supplementary material for: High genetic diversity in the offshore island populations of the tephritid fruit fly Bactrocera dorsalis
Source: BMC Ecol. 2016 Oct 13;16:46. doi: 10.1186/s12898-016-0101-0 (PMC5062917; doi:10.1186/s12898-016-0101-0)
Supplement: Supplementary file 1 — Additional file 1: Table S1. Primer sequences and PCR characteristics of 8 microsatellite loci. Table S2. Indices of genetic diversity and gene flow inferred from microsatellite data. [file 12898_2016_101_MOESM1_ESM.docx]

**High genetic diversity in the offshore island populations of the tephritid fruit fly *Bactrocera dorsalis***

Chunyan Yi^1#^, Chunyan Zheng^2#^, Ling Zeng^3^*, Yijuan Xu^4^*

1 Email: 604472041@qq.com, Laboratory of Insect Ecology, Department of Entomology, South China Agricultural University, Guangzhou 510640, China

2 Email: chunyanzheng01@yahoo.com, Laboratory of Insect Ecology, Department of Entomology, South China Agricultural University, Guangzhou 510640, China

3 Email: zengling@scau.edu.cn, Laboratory of Insect Ecology, Department of Entomology, South China Agricultural University, Guangzhou 510640, China

4 Email: xuyijuan@scau.edu.cn, Laboratory of Insect Ecology, Department of Entomology, South China Agricultural University, Guangzhou 510640, China

#Chunyan Yi and Chunyan Zheng have contributed equally to this work.

∗ Correspondence to: Yijuan Xu & Ling Zeng, Department of Entomology, College of Agriculture, South China Agricultural University, Guangzhou 510640, China. E-mail: [xuyijuan@yahoo.com](mailto:xuyijuan@yahoo.com)

**Supplementary Information**

Table S1 Primer sequences and PCR characteristics of 8 microsatellite loci

| Locus | Repeat motif | Primer sequence (5' to 3') | Product (bp) | | Tm (°C) |
| --- | --- | --- | --- | --- | --- |
| Ccmic32 | ( TTG) _n_ (GTG)_m_ (ATG) _i_ | ACCACCCAATAACTTCATA GCTTTCATCATCCGTTCC | 184 | 52 | |
| 6.8A | ( CAT) _5_ | AGCAACACTATAGGCTGGTC GTAACGCGTTACTGTCATTG | 147 | 53 | |
| 4.6A | （GT) _8_ | CATGCATGTGACAAGGAGA ATGTACAGCCGAGGTAAATG | 137 | 54 | |
| Bo-D48 | ( CA) _13_ | GCCATGAATGCAGACCAC TATTCAAATGCACGCAAAAC | 152 | 54 | |
| 4.3A | ( TTG) _6_TGG( TTG) _3_ | TATGCTGCGTCGCTTATACC AAGTGCTTTAACTGCGTTCG | 94 | 54 | |
| MS4 | (AGCGAC)_3_(AGCAAC)_2_ | CTTGATTGCACCGCGCTTACCC CTCATGCACTACGCGGCCATTCG | 169 | 57 | |
| MS6 | (GTCT)_2_(GTCC)(ATCT)(GTCT)(GTAT) | ATCAGCATGACGATCAGAGTTGA TGTACAGTTGCCGTGGACAATGC | 193 | 58 | |
| MS12A | (AG)_2_AA(AG)_4_ | CCCATGCACTGAGGCACGAAG GTCAAGCGTTTGACGCTCTTCAG | 216 | 58 | |

Table S2 Indices of genetic diversity and gene flow inferred from microsatellite data

| Location | *N_A_* | *N_E_* | *I* | *H_O_* | *H_E_* | *F_IS_* | *F_IT_* | *F_ST_* | *N_em_* |
| --- | --- | --- | --- | --- | --- | --- | --- | --- | --- |
| 6.8A | 19 | 8.1241 | 2.2974 | 0.6417 | 0.8806 | 0.2367 | 0.2683 | 0.0414 | 5.7919 |
| MS6 | 12 | 2.1622 | 1.1702 | 0.1167 | 0.5397 | 0.7506 | 0.7829 | 0.1298 | 1.6754 |
| MS12 | 13 | 4.8411 | 1.8582 | 0.1833 | 0.7968 | 0.7406 | 0.7689 | 0.1091 | 2.0415 |
| 4.3A | 7 | 2.4926 | 1.1207 | 0.1667 | 0.6013 | 0.7014 | 0.7217 | 0.0680 | 3.4288 |
| Ccmic/ | 12 | 2.8328 | 1.4329 | 0.1008 | 0.6497 | 0.8353 | 0.8423 | 0.0420 | 5.6975 |
| Bo-D48 | 15 | 6.4043 | 2.1682 | 0.5250 | 0.8474 | 0.3342 | 0.3779 | 0.0655 | 3.5640 |
| 4.6A | 16 | 6.9331 | 2.1996 | 0.4167 | 0.8593 | 0.4891 | 0.5131 | 0.0469 | 5.0800 |
| MS4 | 13 | 3.2976 | 1.5186 | 0.6441 | 0.6997 | 0.0365 | 0.0750 | 0.0399 | 6.0098 |
| mean | 13.375 | 4.636 | 1.7207 | 0.3494 | 0.7343 | 0.4884 | 0.522 | 0.0656 | 3.5605 |

N_A_: Observed number of alleles; N_E_: Effective number of alleles [42]; *I*: Shannon's information index [43]; H_O_: Observed heterozygosity; H_E_: Expected heterozygosity; F_IS_: Inbreeding coefficient within a population; F_IT_: Total inbreeding coefficient; F_ST_: Differentiation coefficient among populations; N_em_: Gene flow among populations
